# Common Peak Approach Using Mass Spectrometry Data Sets for Predicting the Effects of Anticancer Drugs on Breast Cancer

**Published:** 2007-12-14

**Authors:** Masaru Ushijima, Satoshi Miyata, Shinto Eguchi, Masanori Kawakita, Masataka Yoshimoto, Takuji Iwase, Futoshi Akiyama, Goi Sakamoto, Koichi Nagasaki, Yoshio Miki, Tetsuo Noda, Yutaka Hoshikawa, Masaaki Matsuura

**Affiliations:** 1 Genome Center, Japanese Foundation for Cancer Research, Tokyo, Japan; 2 Cancer Institute, Japanese Foundation for Cancer Research, Tokyo, Japan; 3 Cancer Institute Hospital, Japanese Foundation for Cancer Research, Tokyo, Japan; 4 Institute of Statistical Mathematics, Tokyo, Japan; 5 Department of Computer Science and Communication Engineering, Kyushu University, Fukuoka, Japan; 6 International University of Health and Welfare Mita Hospital, Tokyo, Japan

## Abstract

We propose a method for biomarker discovery from mass spectrometry data, improving the common peak approach developed by Fushiki et al. (*BMC Bioinformatics*, 7:358, 2006). The common peak method is a simple way to select the sensible peaks that are shared with many subjects among all detected peaks by combining a standard spectrum alignment and kernel density estimates. The key idea of our proposed method is to apply the common peak approach to each class label separately. Hence, the proposed method gains more informative peaks for predicting class labels, while minor peaks associated with specific subjects are deleted correctly. We used a SELDI-TOF MS data set from laser microdissected cancer tissues for predicting the treatment effects of neoadjuvant therapy using an anticancer drug on breast cancer patients. The AdaBoost algorithm is adopted for pattern recognition, based on the set of candidate peaks selected by the proposed method. The analysis gives good performance in the sense of test errors for classifying the class labels for a given feature vector of selected peak values.

## 1. Introduction

Recent technological innovation has brought us comprehensive methods for the analysis of protein expression profile data, such as Surface-Enhanced Laser Desorption/Ionization Time of Flight (SELDITOF) or Matrix-Assisted Laser Desorption/Ionization (MALDI)-TOF mass spectrometry (MS). After initial research on the early detection of ovarian cancer (Petricoin et al. 2002), new methodologies for data analyses have been developed (Yasui et al. 2003; Tibshirani et al. 2004; Geurts et al. 2005; Meleth et al. 2005; Yu et al. 2005). As the TOF data has both amplitude variation and phase variation (Lin et al. 2005), there are ongoing discussions about analytical problems (Conrads et al. 2004; Lyons-Weiler et al. 2005). Briefly, the issue of analytical approaches includes the alignment and detection of peaks, and the construction of classifiers for phenotypes. In the methods of detecting peaks, searching local maxima with a local signal-to-noise ratio is the most often used method. However the methods using wavelet transformation (Qu et al. 2003, etc.) were used as well. Recently, Fushiki et al. (2006) proposed the common peak method identifying biomarkers from high-dimensional MS data. This idea is based on the observation that peaks shared with for only few subjects may be noise, whereas peaks shared with more subjects may be significant.

In this paper, we investigated the performance of the common peak method. We applied the common peak method to each group of subjects by class label rather than to the entire group, as by Fushiki et al. (2006). We applied this proposed approach to a SELDI-TOF MS data set from samples of cancer tissue obtained by laser capture microdissection (LCM) before the patients received medication with anticancer drugs. We examined the data set to detect effective peaks and constructed a system for predicting the effects of neoadjuvant therapy with an anticancer drug on breast cancer patients. Neoadjuvant therapy is one of the breast-conserving therapies that uses anticancer drugs before surgery. After the cancer has been reduced in size, surgical excision can be applied. An establishment of prediction systems of the treatment effect of an anticancer drug (e.g. a system predicting a patient being responder or nonresponder) would improve decision making on each patient’s best medication. Such prediction systems could prevent useless medication and could help in selecting appropriate personalized therapies. We used the data set for illustrative purposes.

The common peak method allows the use of continuous and discrete covariates for peak intensities. In our study, we also examined both types of covariates in the analyses of statistical testing for each selected common peak and in the construction of a prediction system by using AdaBoost (Freund and Schapire, 1997) which is known as one of the efficient ensemble learning methods. We found that the classifier learning from discrete covariates showed very high performance. We discuss below some practical problems of data preprocessing in the mass spectrometry data and provide a guideline for the treatment of these data.

## 2. Data Sets Used

In our study, tissues from 65 breast cancer patients were sampled between 2003 and 2004 from the Cancer Institute Hospital at the Japanese Foundation for Cancer Research (Tokyo, Japan). The samples met all the following eligibility criteria: size ≥ 3.0 cm of invasive cancer; Stage IIA–IIIB; age ≤ 70; bone marrow, liver and kidney functions were maintained (WBC ≥ 4000/mm^3^, Plat. ≥ 100,000/mm^3^, Hb ≥ 10 g/dl, GOT/GPT < 60/70 U/I) and the patients had no other serious complications.

All patients received docetaxel 75 mg/m^2^ four times weekly every three weeks as neoadjuvant chemotherapy before surgery. Fresh cancer tissue biopsies were taken before treatment and cancer cells were isolated by LCM. To ensure the high quality of the samples, LCM was used in proteomic analyses (Verma et al. 2001; Batorfi et al. 2003; Wulfkuhle et al. 2003; Krieg et al. 2005). The treatment effect was judged from the pathology of specimens removed at subsequent surgery. Forty-two patients were classed into a nonresponding group with pathology grade ≤1a (mild response). The other 23 patients with pathology grade ≥1b (moderate response) were classed as a group that responded to treatment. Here the pathological response 1a (mild response) is defined as mild changes in cancer cells regardless of the area, or marked changes seen in less than one third of cancer cells, and 1b (moderate response) is defined as marked changes in one third or more but less than two thirds of tumor cells (The Japanese Breast Cancer Society, 2005). The total set of 65 patients was randomly separated into 50 training samples and 15 test samples, as shown in Table 1.

## 3. Methods

### 3.1. Preprocessing

In MS data, the *x*-axis shown here denotes the time of flight that was transformed into the mass-to-charge ratio (m/z value), and the *y*-axis denotes the intensity. Procedures for preprocessing observed spectra were as follows: (1) baseline subtraction, (2) alignment of mass spectra and (3) normalization. We used SpecAlign software (Wong et al. 2005) for procedures (1) and (2). Generation of spectrum averages and alignment using the peak matching method were performed in procedure (2). Normalization was performed using the method of Baggerly et al. (2004). For a single spectrum, let *V_i_* denote the raw intensity at the *i*-th m/z value, and let *V*_min_ and *V*_max_ denote the smallest and largest observed intensities in the spectrum, respectively. Then the normalized intensity *NV_i_* is given by

$$NV_{i}=\frac{V_{i}-V_{\text{min}}}{V_{\text{max}}-V_{\text{min}}},$$

and all *NV_i_*’s are in the [0, 1] range.

### 3.2. Common peak method

To identify proteins associated with phenotypes, we needed to discriminate between noise and peaks of protein expression in observed spectra. Fushiki et al. (2006) proposed a new method for peak detection using published data. The principle of this method is that peaks may come from protein expression rather than noise when peaks are commonly observed in a major portion of subjects.

Using this method, covariates for prediction were constructed in the following order:

1. Peak detection for each subject
2. Common peak detection among subjects
3. Calculation of discrete and continuous covariates by each subject.

#### 3.2.1. Peak detection for each subject

First, we detected peaks for each subject, using the method proposed by Yasui et al. (2003). We set a *k*-nearest neighborhood as a width of window on the *x*-axis. Here we used *k* = 10. An m/z value achieving the maximum intensity in that window is regarded as a peak. We then moved the window along the *x*-axis and search for peaks in the spectrum of each subject.

#### 3.2.2. Common peak detection among subjects

Next, we constructed common peaks by a responder and a nonresponder group from each individual’s peaks. In this step, we calculated an average of peaks, *A*(*x*), constructed by averaging Gaussian kernels with centers at the individual peak. Then *A*(*x*) was expressed as

(1) $$A(x)=\frac{1}{N_{G}}\sum_{i=1}^{N_{G}}\sum_{j}\text{exp}\left[-\frac{{\left(x-p_{i,j}\right)}^{2}}{{\left(\sigma p_{i,j}\right)}^{2}}\right],$$

where *N_G_* is the sample size of each group, *p_i,j_* is the m/z value of the *i*-th subject’s *j*-th peak and σ is a parameter accounting the width of the peak. We used here σ = 0.001.

**Figure 1.**
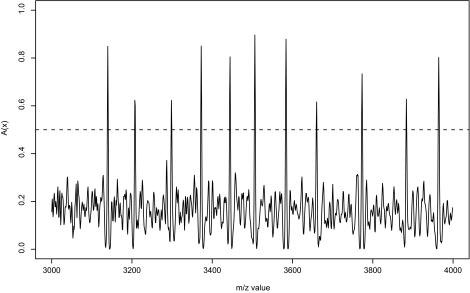
Average of peaks (nonresponders). The dashed line denotes a threshold value *h* = 0.5.

The common peak is defined by the point *x* in which *A*(*x*) is greater than a certain threshold *h*. Figure 1 shows the curve of *A*(*x*) at *x* = [3000*,* 4000] from nonresponders. Here we used *h* = 0.5.

Fushiki et al. (2006) obtained common peaks using all subjects of a study at once, however, in our work we applied the common peak method separately to each of two groups, as our purpose was to detect informative peaks for discrimination between responder and nonresponder. The feature of our approach is that it uses information of labels of groups but it does not intend any discrimination for a particular common peak. If the same common peak is selected for both groups, it would not help in discrimination. However, when a common peak is detected only in one group, then that peak would be an appropriate candidate for classifiers. Below we will compare the proposed method with that by Fushiki et al. (2006).

### 3.3. Calculation of discrete and continuous covariate by each subject

We often analyze data sets with discrete covariates, which are dichotomous codes with 0 and 1 rather than direct intensity when there might be a relative large error of intensity of SELDI and MALDI. In this case, a covariate *x_j_* for the common peak *m_j_* for the *j*-th peak for a certain subject might be obtained as follows:

(2) $$x_{j}=\{\begin{array}{ll} 1, & \text{if}\;\text{there}\;\text{exists}\;\text{a}\;\text{peak}\;\text{within}\\ & \text{a}\;\text{window}\;[(1-\rho )m_{j},(1+\rho )m_{j}],\\ 0, & \text{otherwise}, \end{array}$$

where ρ adjusts a width of the window around the common peak *m_j_*, and should be set according to precision of m/z values. On the other hand, when we aim to use the observed value of intensity, the continuous covariates *x_j_* is defined as follows:

$$\begin{array}{l} x_{j}=\text{the}\;\text{maximum}\;\text{value}\;\text{of}\;\text{the}\;\text{intensity}\;\text{within}\\ \;\;\;\;\;\;\;\;\;\;[(1-\rho )m_{j},(1+\rho )m_{j}]. \end{array}$$

In this study, we employed both cases of discrete and continuous covariates with ρ = 0.0005.

### 3.4. AdaBoost

AdaBoost is one of the machine learning algorithms which is ensembles of statistical classifiers that are more accurate than a single classifier. It is known that the boosting algorithm is highly resistant to overfitting in the discovery of protein biomarkers (Yasui et al. 2003).

We consider here a set of the training data set *D =* {(*x_i_*, *y_i_*):*i* = 1, …, *N*}, where *x* is an input vector and *y* ∈ {+1 −1} is a class label. In this paper *x* corresponds to a set of covariates based on the common peaks. Let

$$F=\{f_{j}(x):j\in \{1,\ldots ,J\}\}.$$

be a set of weak classifiers. Here *J* is the total number of common peaks among groups. Then the AdaBoost algorithm is described as follows:

1. Set an initial value of weight *w_1_* (*i*) *=* $\frac{1}{N}$, (*i* = 1, … *, N*).
2. Define a weighted error rate for *t*-th iteration, *t* = 1*,* … *, T*, as
  (3) $$\varepsilon_{t}(f)=\sum_{i=1}^{N}I(f(x_{i})\neq y_{i})w_{t}(i)$$
  where *I* represents indicator function and *w_t_* is a weight at *t*-th iteration. Next,
  (2a) Select a weak classifier *f_t_* = argmin_1_*_≤ j≤ J_* ɛ*_t_*(*f_j_*).
  (2b) Calculate $\alpha_{t}=\frac{1}{2}\text{log}\frac{1-{ɛ}_{t}(f_{t})}{{ɛ}_{t}(f_{t})}$.
  (2c) Update the weight defined by *w_t_*_+1_(*i*) ∝ *w_t_*(*i*) exp {−*y_i_α_t_* *f_t_* (*x_i_*)}. Here we normalize the weight such that ∑*_i_* *w_t_*_+1_(*i*) = 1.
3. *f_T_* (*x*) = sgn(*F_T_*(*x*)), where $F_{T}(x)=\sum_{t=1}^{T}\alpha_{t}f_{t}(x)$.

In step (2a), *f_t_* is adjusted for a restriction that the weighted error rate must be less than 0.5. If it exceeds 0.5, then we use −*f_t_* instead of *f_t_* as a classifier. Furthermore, step (2c) can be expressed as:

(4) $$w_{t+1}(i)\;\;\propto \{\begin{array}{cc} w_{t}(i)\text{exp}(\alpha_{t}), & \text{if}\;\;f_{t}(x_{i})\neq y_{i}\\ w_{t}(i)\;\text{exp}(-\alpha_{t}), & \text{if}\;f_{t}(x_{i})=y_{i} \end{array}.$$

We update the weight by multiplying with exp(α*_t_*) when *f_t_* misjudges the *i*-th subject, and by multiplying with exp (−α*_t_*) when *f_t_* judges correctly the *i*-th subject (see Murata et al. 2004).

We adopted cross-validation (CV) for selecting the number of classifiers *T* as follows: We select the minimum of *T* such that the integrated classifier *f_T_* in step 3 attains local minima and has CV errors with no more than one standard error above the minimum CV error (see Hastie et al. 2001).

## 4. Results

### 4.1. Common peak detection

From the training data set, we obtained 92 common peaks for the responder group of 18 patients and 81 common peaks for the nonresponder group of 32 patients. All common peaks which were detected for at least one group were used for analysis. In total, 117 common peaks were obtained. We calculated both discrete and continuous covariates for these 117 common peaks.

### 4.2. Construction of classifiers

**Figure 2.**
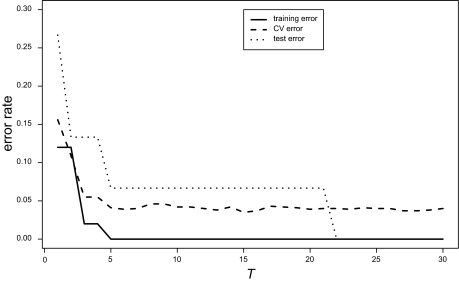

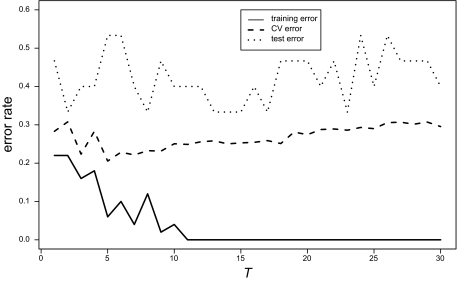
Training error rate (solid line), CV error rate (dashed line), and test error rate (dotted line) by AdaBoost for the discrete and continuous covariates. **(a) discrete covariates** **(b) continuous covariates**

To construct a classifier, we analyzed the training data set using AdaBoost and computed the training and CV errors. CV error was calculated by replicating a five-fold cross-validation 50 times and averaging the errors. Figure 2(a) shows the error curves of the discrete case. The CV error (dashed line) was minimized locally at *T* = 6 and the error rate at *T* = 6 did not differ statistically significant from that of the best model (*T* = 15). Therefore, we selected the six-peaks model for the discrete case.

The error curves of the continuous case with normalization are shown in Figure 2(b), but the CV error rates for entire range of *T* were much worse than that for the discrete covariates. Therefore there were not any comparable model for the continuous case.

### 4.3. Validation result

**Figure 3.**
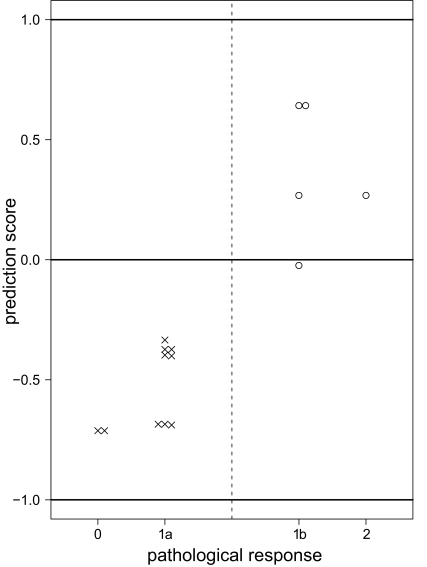
Prediction scores of the test data for discrete covariates using six-peaks model. The “○” indicate the five responders and the “×” indicate the ten nonresponders. Only one of the subjects with response “1b” is misclassified.

Using the six-peaks model, we predicted treatment effects, (i.e. “responder or nonresponder”), for each subject in the test data of 15 subjects. The test error was 1/15 for the discrete covariates (Fig. 2(a)). Figure 3 shows the prediction scores for all subjects of the test data using discrete covariates. The prediction score *F*′(*x*) is given by

(5) $$F^{\prime }(x)=\frac{\sum_{t=1}^{T}\alpha_{t}f_{t}(x)}{\sum_{t=1}^{T}\alpha_{t}}.$$

This score has the property that when the score is more distant from the zero value, the prediction is more confident.

### 4.4. Single peak analysis

For selecting six peaks by AdaBoost, we performed Fisher’s exact test to investigate whether the frequency of the peak differed between groups. Table 2 indicates that the result was significant for all six peaks.

### 4.5. Comparison with the original common peak method

To compare it with the original common peak method, we analyzed the same data set using the unsupervised method of Fushiki et al. (2006). This method detected 81 common peaks from 50 samples of the training data set, but there were no differences in the frequencies of peaks between responders and nonresponders. AdaBoost was also applied but the training error rate could not be forced to become zero. It was not possible to discriminate the training data set sufficiently when using the original common peak method. From this we conclude that the new method is superior for our task.

### 4.6. Comparison with another classification approach

It is important to compare this method with other classification methods such as the support vector machine-recursive feature elimination (SVM-RFE) approach (Guyon et al. 2002). We re-analyzed our data using the SVM-RFE with polynomial and Gaussian kernels instead of AdaBoost. In the discrete case, the fifteen peaks model with 2nd degree polynomial kernel was selected by the RFE. The five-fold CV error of this model was 0.024 and the test error was 2/15. Only three peaks (m/z values 1361, 2250 and 2989) selected by AdaBoost were included in the fifteen peaks model. The SVM-RFE approach gave comparable results, but we concluded AdaBoost was better for our analysis in that the number of peaks in the model was small and the test error was small.

## 5. Discussion

In the common peak method proposed here, one has to set four parameters (*k, h,* ρ, σ) to select common peaks and give the individual covariates. If the parameter *k* concerning window width is small, as the probability of selecting false positives is high and hence the baseline of average peaks is also high. We adopted *k* = 10 following the original method of Fushiki et al. (2006); we also tried *k* = 20, but the resulting common peaks showed no difference. The threshold parameter *h* was used for detection of the common peaks.

When the sample size *N_G_* is small in equation (1), the impact of uncommon peaks on *A*(*x*) is large. Therefore in such a case *h* should also be large. Parameters ρ and σ, should be set to properly account for the width of the peak, because it is difficult to align spectra perfectly in the stage of preprocessing. SELDI-TOF machine we used has an error of 0.002 about its m/z values, and ρ and σ should be less than 0.002 because the spectrum alignment had been performed already. In our study, we set ρ = 0.0005 and σ = 0.001 for this reason.

In our study, we realized that the result for continuous covariates was worse than that for discrete covariates. Continuous covariates are calculated based on the intensity with baseline subtraction. Hence they become sensitive to the variation caused by the methods or parameter settings of baseline subtraction. In contrast, discrete covariates are not influenced by baseline subtraction. In this sense, we supported the results based on the case of discrete covariates in this analysis.

On the use of the common peak method, if the spectra alignment is insufficient, the number of detected common peaks becomes smaller, and consequently it may fail to detect important peaks. Therefore, preprocessing—especially for spectra alignment—is important when analyzing MS data.

It is important to compare the common peak method with other peak selection methods. We analyzed our data using the Ciphergen ProteinChip software (Fung and Enderwick, 2002) with default settings, 82 peaks of 117 peaks obtained by our method were detected from 50 training samples, and only three peaks (m/z values 1361, 2250 and 2621) were detected of the six peaks we selected as the biomarkers. ProteinChip software judges whether an intensity is a peak caused by protein expression or noise for each single spectrum. Then peak alignment after the peak detection is needed for classification. On the other hand, the common peak method detects peaks from multiple spectra, and hence our method can detect peaks more sensitively.

In order to see how the results would be affected if different training and test sets were used, we examined randomly splitting the spectra into training and test 10 times. The mean of the number of selected peaks was 7.4 and its range was 4 to 12. Among the six peaks selected in Section 4.2, the peak with m/z value 2843 was selected in all 10 cases, and the other peaks were selected at least three cases. The mean test error was 0.087, and this error rate had little difference from 1/15(=0.067). Therefore it was confirmed that our result did not depend on the choice to split training and test sets.

We compared the proposed method with the SVM-RFE method in Section 4.6 and concluded that AdaBoost was slightly better than the SVM-RFE in our analysis, but there was no significant difference. However, the SVM-RFE method has more computational complexity, because the SVM-RFE learns using all variables first to compute the ranking criterion for all variables, and removes the least important variable in a sequential manner. On the other hand, AdaBoost minimizes sequentially the exponential loss function and selects important variables simultaneously. AdaBoost has an advantageous point from the computational reason with an appropriate stopping rule.

Validating common peaks obtained here on other available breast cancer data sets is important. Pusztai et al. (2004) used SELDI-TOF MS profiling to examine proteomic changes in plasma of patients with breast carcinoma who received either preoperative or postoperative chemotherapy for Stage I–III breast carcinoma. They detected only one treatment-induced protein/peptide peak (m/z value 2790) and reported five peaks (m/z values 3165, 3440, 4115, 4444, and 8940) that expressed in plasma obtained from women breast carcinoma. Among the common peaks obtained in our study, one peak with m/z value 3444 was close to one of the peaks (m/z value 3440) reported by Pusztai et al. (2004). This peak was observed in both case and control groups as a common peak. However, it is hard to confirm the consistency, because there are many differences between two studies compared. We used microdisected cancer tissue, but Pusztai et al. (2004) used plasma and they used paclitaxel chemotherapy or 5-fluorouracil, doxorubicin, and cyclophosphamide (FAC) chemotherapy. Therefore, further examination will be needed to validate these peaks and biomarkers obtained here.

## Figures and Tables

**Table 1.** Pathologies of the 65 patients. The effects 0 and 1a are defined as nonresponders; 1b and 2 are defined as responders.

**Group**	**Nonresponder**		**Responder**		

**Pathology effect**	**0**	**1a**	**1b**	**2**	**Total**
Training	4	28	11	7	50
Test	2	8	4	1	15
Total	6	36	15	8	65

**Table 2.** *p*-values of the single peak analysis.

**Group**	**Responder**		**Nonresponder**		

**m/z value**	**Peak**	**Nonpeak**	**Peak**	**Nonpeak**	***p*-value**
1361	10	8	32	0	8.15E–5
2250	17	1	13	19	2.00E–4
2621	16	2	12	20	8.11E–4
2843	3	15	29	3	2.28E–7
2989	17	1	12	20	7.37E–5
6557	9	9	30	2	6.84E–4
